# Impairment of root auxin–cytokinins homeostasis induces collapse of incompatible melon grafts during fruit ripening

**DOI:** 10.1093/hr/uhac110

**Published:** 2022-05-17

**Authors:** Maria Dolores Camalle, Aleš Pěnčík, Ondřej Novák, Lina Zhao, Udi Zurgil, Aaron Fait, Noemi Tel-Zur

**Affiliations:** 1 The Albert Katz International School for Desert Studies, The Jacob Blaustein Institutes for Desert Research, Ben-Gurion University of the Negev, Sede Boqer Campus 8499000, Israel; 2 Laboratory of Growth Regulators, Faculty of Science, The Czech Academy of Sciences, Palacký University & Institute of Experimental Botany, Šlechtitelů 27, CZ-783 71 Olomouc, Czech Republic; 3 French Associates Institute for Agriculture and Biotechnology of Drylands, Blaustein Institutes for Desert Research, Ben-Gurion University of the Negev, Sede Boqer Campus, P.O.B. 653, Beer Sheva 84104000, Israel

## Abstract

The factors underlying the plant collapse of certain melon-pumpkin graft combinations are not fully understood. Our working hypothesis was that impairment of photoassimilates transport in incompatible combinations induces an imbalance in the homeostasis of root auxin (indole-3-acetic acid; IAA) and of cytokinins, probably triggering plant collapse. Root IAA and cytokinins levels in the presence and absence of fruit and changes in root and scion metabolites were investigated in compatible and incompatible combinations. We showed elevated levels of IAA, 2-oxoindole-3-acetic acid (IAA catabolite), indole-3-acetylaspartate (IAA conjugate), and *cis*-zeatin-type cytokinins, but low levels of *trans*-zeatin-type cytokinins in the roots of plants of the incompatible combination during fruit ripening. Similarly, during fruit ripening, the expression of the *YUCCA* genes, *YUC2*, *YUC6,* and *YUC11* (required for auxin biosynthesis), the *GRETCHEN-HAGEN3* gene (required for auxin conjugation), and the *cytokinin oxidase/dehydrogenase 7* (*CKX7*) gene (regulates the irreversible degradation of cytokinin) was enhanced in the roots of plants of the incompatible combination. Moreover, in the incompatible combination the fruiting process restricted transport of photoassimilates to the rootstock and induces their accumulation in the scion. In addition, high levels of hydrogen peroxide and malondialdehyde and reduced activity of antioxidant enzymes were observed in the roots of the incompatible graft. Our results showed that the collapse of the incompatible graft combination during fruit ripening is closely associated with a dramatic accumulation of IAA in the roots, which probably elicits oxidative damage and disturbs the balance of IAA and cytokinins that is of critical importance in melon-pumpkin graft compatibility.

## Introduction

The grafting of melon (*Cucumis melon* L.) onto pumpkin (*Cucurbita maxima* Duch. × *Cucurbita moschata* Duch.) rootstock has been used to overcome cultivation challenges and to improve melon yield [1–4]. However, scion-rootstock incompatibility remains a major bottleneck, limiting the widespread implementation of interspecific grafting by melon growers [5–8]. Graft incompatibility between melon scion and pumpkin rootstock, which can manifest itself at any time of the grafting cycle life, may be attributed to a variety of physiological and biochemical factors [6–9], such as oxidative stress at the graft junction or accumulation of auxin (indole-3-acetic acid; IAA) in the root, which trigger plant collapse a few days after grafting (DAG) [9–11].

It is currently held that the main triggers that induce the collapse of incompatible melon-pumpkin combinations during late stages of the grafted plant’s life, especially during fruit ripening, are increased water stress (resulting from a heavy fruit load) and/or the inhibition of photoassimilate transport from the scion to the rootstock due to a blockage in the graft zone [6–8]. However, the recent investigation of Camalle et al. [12] of photoassimilate trafficking from the scion to the rootstock in different compatible and incompatible melon-pumpkin combinations showed that specific metabolites (mainly amino acids, sugars and sugar alcohols) accumulated in the scion of the incompatible combination during fruit maturation, even though there was no evidence of a blockage in the graft zone [12]. Moreover, relatively high levels of *cis*-zeatin-type (*c*Z-type) cytokinins and IAA (both synthesized mainly in the leaves) were observed only in the rootstock of the incompatible combination—a finding that also negates the premise of a blockage in the graft zone [12]. Therefore, the increased accumulation of the above-mentioned metabolites in the scion sap of the incompatible combination could not have been the sole trigger for the plant collapse. Camalle et al. [12] also reported lower levels of *trans*-zeatin-type (*t*Z-type) cytokinins in the rootstock of the incompatible combination. This finding suggests that a delay in the transport of photoassimilates from the scion to the rootstock could compromise the production in the roots of the biologically important *t*Z-type cytokinins [13, 14], with the decreased production having the potential to impair leaf growth [15, 16]. A number of studies have reported that both *t*Z-type and *c*Z-type cytokinin profiles are affected by stress conditions. For example, elevation of the concentration of *cis*-zeatin riboside (*c*ZR) cytokinins with a simultaneous decline of *trans*-zeatin riboside (*t*ZR) cytokinins has been found in the leaves and roots of tobacco plants in the early response to heat stress [17], in the roots of maize plants after exposure to salt stress [18], and in the roots of tobacco plants exposed to drought stress [19]. It is thus likely that the stress induced by a lack of graft compatibility will also affect *t*Z-type and *c*Z-type cytokinin profiles in grafted plants; in such cases, the rootstock genotype would probably be the main factor responsible for inducing the stress.

As mentioned above, Camalle et al. [12] reported higher IAA concentrations in the rootstock sap of an incompatible combination than in self-grafted plants, which probably suggests impairment of IAA synthesis. The ability of plants to cope with extreme IAA concentrations is manifested via the major pathway for auxin inactivation, comprising the fast oxidation of IAA to 2-oxindole-3-acetic acid (oxIAA) [20–23] and the conjugation of IAA into indole-3-acetic acid aspartic acid (IAAsp) and indole-3-acetic acid glutamic acid (IAGlu) [23–26]. At the molecular level, the two mechanisms that control IAA homeostasis are IAA synthesis, which is modulated mainly by the family of *YUCCA* (*YUC*) genes [27–29], and IAA degradation into amino acids, which is controlled by the family of *GRETCHEN-HAGEN3* (*GH3*) genes [26]. Therefore, the accumulation of IAA observed in the roots and rootstock sap of incompatible melon graft combinations [7, 10, 12] indicated that IAA synthesis and/or degradation and conjugation pathways could be impaired in incompatible graft combinations. In light of the concerted interaction of IAA and cytokinins in controlling plant growth [30], the alterations found by Camalle et al [12] in the endogenous plant hormone status in the incompatible melon-pumpkin graft combination, i.e. increased IAA levels and decreased total *t*Z-type-cytokinins in the rootstock sap, constitute empirical evidence of a hormonal imbalance—one that probably induced plant collapse during ripening [12]. Therefore, there are two possible explanations for the elevated IAA concentrations in the rootstock sap of the incompatible melon-pumpkin graft—either enhanced IAA transport from the scion to the rootstock or increased synthesis of IAA in the root. Whatever the explanation, it is likely that the resultant accumulation of IAA in the roots will affect the synthesis of *t*Z-type-cytokinins in incompatible melon-pumpkin grafted plants during fruit ripening, resulting in plant collapse.

Taking our findings together with previous work published on the subject, our working hypothesis for this study was that the plant collapse of incompatible melon-pumpkin combinations during fruit ripening is due to fruit demands for photoassimilates, leading to the following sequence of events: a reduction in the level of photoassimilates reaching the roots will lead to a decrease in the synthesis of *t*Z-type cytokinins and an increase in IAA synthesis (via the upregulation of *YUC* genes to promote root growth), which, in turn, will lead to impairment of the root IAA–cytokinins homeostasis. These changes are likely to induce the production of reactive oxygen species (ROS), leading to the accumulation of malondialdehyde (MDA) and, ultimately, to rootstock senescence and plant collapse. Therefore, this study set out to investigate the homeostasis of IAA and of cytokinins during fruit ripening in compatible and incompatible melon-pumpkin graft combinations, with a self-grafted melon serving as the control. To this end, hormone and metabolomic profiling, gene expression monitoring, and biochemical analysis of the sap of roots and/or leaves of compatible and incompatible melon-pumpkin grafted and self-grafted melon plants were performed under controlled conditions in an experiment involving one of two treatments, either fruiting or fruit removed. An additional experiment followed changes in root hormone homeostasis and in the contents of stem metabolites in incompatible melon-pumpkin grafts during graft collapse.

## Results

### Leaf hormone and metabolomic profiling reveals minor differences between graft combinations and between treatments

The melon-pumpkin graft combinations used in this study comprised “Kiran” (designated Ki) melon scion grafted onto compatible “TZ-148” (designated TZ) and incompatible “53 006” (designated r53) pumpkin rootstocks. Self-grafted Ki was used as the control. Leaf hormone levels and the profile of metabolites were examined at three plant stages, vegetative, flowering, and fruit ripening (when the first fruit turned yellow and the collapse of incompatible combination began).

**Figure 1:**
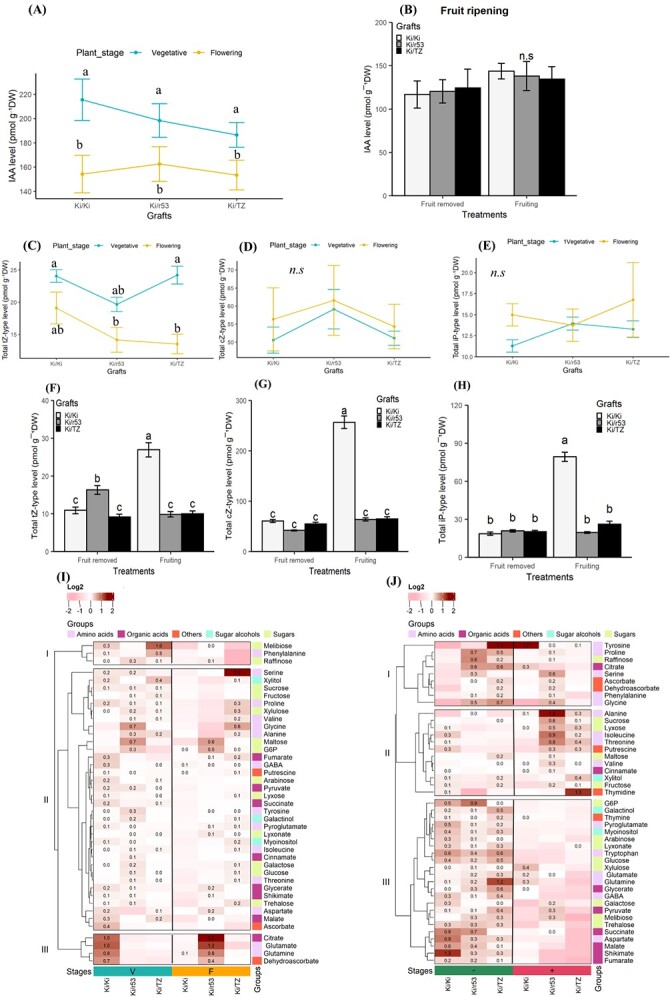
**Comparison of leaf metabolites between graft combinations and between treatments, i.e. fruiting and fruit removed.** (A) Leaf auxin (IAA) content in self-graft (Ki/Ki) and heterograft (Ki/r53 and Ki/TZ) combinations at the vegetative and flowering stages. (B) Leaf auxin (IAA) content at fruit ripening stage in fruiting and fruit removed plants. (C–E) Contents of total leaf *t*Z-type cytokinins (C), *c*Z-type cytokinins (D), and iP-type cytokinins (E) at the vegetative and flowering stages. (F–H) Contents of total leaf *t*Z-type cytokinins (F), *c*Z-type cytokinins (G), and iP-type cytokinins (H) at the fruit ripening stage. (I) Heat map showing the hierarchical clustering of the metabolite log_2_ values at two stages, vegetative (V) and flowering (F). (J) Heat map showing of leaf metabolites at the fruit ripening stage for fruit removed (−) and fruiting (+) plants. For (A–H) data are means ± se (*n* = 4 biological replicates). Different lowercase letters indicate significant differences evaluated by the Tukey–Kramer multi-comparison test conducted only when a two-way analysis of variance was significant at *p* < 0.05. For (I, J), dark red in the heat maps indicates a high relative abundance of metabolites, and light pink indicates a low relative abundance.

At the vegetative and flowering stages, the leaf IAA concentrations showed no differences between the graft combinations (Fig. 1A). Similarly, no differences were found between graft combinations or treatments (fruit removed and fruiting) at the fruit ripening stage (Fig. 1B). Moreover, levels of leaf *t*Z-type, *c*Z-type, and N^6^-isopentenyladenine type (iP-type) cytokinins did not differ between graft combinations at the vegetative and flowering stages (Fig. 1C–E). In contrast, in the presence of fruit, the contents of leaf *t*Z-type, *c*Z-type, and iP-type cytokinins were significantly increased in the self-grafted plants vs. the two heterograft combinations, Ki/TZ and Ki/r53, in which the levels of all the cytokinins remained unchanged, irrespective of the treatment (Fig. 1F–H). The only exception was a decrease in *t*Z-type cytokinins in the leaves of Ki/r53 in the presence of fruit (Fig. 1F).

As presented in the heat maps, the relative abundances leaf metabolites were determined at the vegetative, flowering, and fruit ripening stages. The most significant differences at the vegetative stage (denoted V in Fig. 1I) were found in the relative abundances of citrate, glutamate, glutamine, and dehydroascorbic acid, which were higher in the self-grafted Ki/Ki plants than in the two heterografts, for which the levels of these four metabolites were similar (Fig. 1I; cluster III). At the flowering stage (denoted F), higher relative abundances of citrate, glutamate, glutamine, and dehydroascorbate were found for Ki/r53 than for Ki/Ki and Ki/TZ (Fig. 1I; cluster III). In contrast, there was a significant difference in metabolites in leaves collected at the fruit ripening stage between fruit removed (colored green and marked with a minus sign) and fruiting plants (colored red and marked with a plus sign) (Fig. 1J). For example, the abundance of nine metabolites in cluster I decreased in the fruiting Ki/TZ plants vs. fruit removed (Fig. 1J). Conversely, in cluster II, the relative abundance of 11 metabolites (mainly alanine, sucrose, isoleucine, and threonine) was high in Ki/53 fruiting vs. fruit removed (Fig. 1J). While the other 24 metabolites showed a significant reduction vs. fruit removed plants for all three graft combinations (cluster III), with the exceptions of xylulose, glutamate, and glutamine (slightly high in Ki/Ki, Fig. 1J). These results indicated that the rootstock genotype affects the accumulation of leaf metabolites according to plant physiological stages; nonetheless, we could not detect a clear indicator, namely, a metabolite marker, for graft incompatibility in the scion.

### Early root senescence during fruit ripening characterizes the incompatible graft

**Figure 2:**
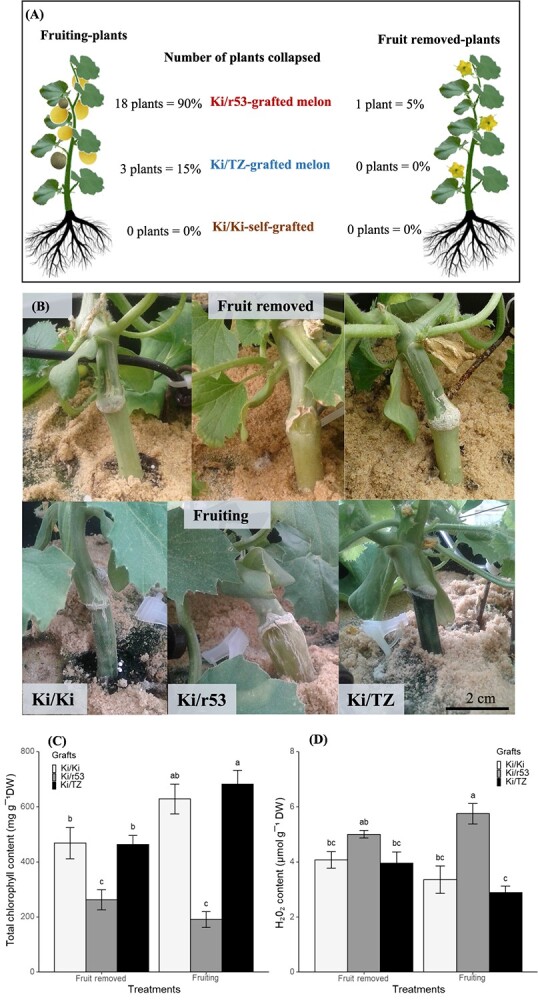
**Comparison between graft combinations and treatments, i.e. fruiting and fruit removed.** (A) Schematic representation of the number of plants that collapsed during the fruit ripening stage. (B) Photographs showing the yellow phenotype of the rootstock (below the graft junction) and grafted parts in self-grafted (Ki/Ki) and heterografted (Ki/r53 and Ki/TZ) plants during fruit ripening, bar represents 2 cm. (C) Total chlorophyll content in the rootstock. (D) H₂O₂ content in the rootstock. Chlorophyll and H₂O₂ data are means ± se (*n* = 4 biological replicates). Different lowercase letters indicate significant differences evaluated by the Tukey–Kramer multi-comparison test conducted only when a two-way analysis of variance was significant at *p* < 0.05.

In our quest to find a marker of graft incompatibility, grafts were visually monitored once a day. The numbers of collapsed Ki/TZ, Ki/r53, and self-grafted Ki/Ki plants in the two treatments, namely, fruiting vs. fruit removed, were monitored over the course of a growing season, May 2018 to September 2018 (Fig. 2A). In the presence of fruit, about 90% of the incompatible Ki/r53 grafted plants and 15% of the compatible Ki/TZ plants collapsed (Fig. 2A). In contrast, only 5% of the Ki/r53 plants and none of the Ki/TZ plants collapsed for the plants whose fruit had been removed. Of note, none of the self-grafted Ki/Ki plants collapsed, regardless of the treatment (Fig. 2A).

During fruit ripening (35 to 40 days after anthesis, when the fruit turned to yellow; see Supplementary Fig. 1), only the rootstocks of the incompatible Ki/r53 grafted plants became yellow and showed the typical progressive signs of senescence until the collapse of the plants at the fruit ripening stage (Fig. 2B). Notably, in the plants of the fruit removal treatment group, the rootstocks of the incompatible Ki/r53 plants become partially yellow, but the plants did not collapse, thereby providing evidence that fruiting contributes to plant collapse. It was also found that during fruit ripening, total chlorophyll content in the leaves of the compatible Ki/TZ plants was higher than that in the incompatible Ki/r53 and self-grafted Ki/Ki leaves, but in the absence of fruit, no differences in total leaf chlorophyll content were observed between the graft combinations (Supplementary Fig. 2).

Following the observations of healthy leaves but premature rootstock senescence in the incompatible Ki/r53 combination, we measured total chlorophyll and H_2_O_2_ contents in the rootstock stem (below the graft junction) of the three combinations. Total chlorophyll content was significantly lower in the rootstocks of the incompatible Ki/r53 grafts than in the compatible Ki/TZ and self-grafted Ki/Ki plants regardless of the treatment (Fig. 2C). No statistically significant differences in rootstock total chlorophyll content were observed in self-grafted Ki/Ki in the presence and absence of fruit (Fig. 2C). However, in the Ki/TZ combination, a statistically significantly increased level of total chlorophyll was observed in the rootstock in the presence of fruit (Fig. 2C). In addition, the H_2_O_2_ content was significantly higher in the rootstock of the incompatible Ki/r53 grafts in the presence of fruit (Fig. 2D), but no statistically significant differences were observed in the three graft combinations between the fruiting and fruit removal treatments (Fig. 2D). Taken together, these findings showed that symptoms of graft incompatibility are visible mainly in the rootstock and not in the scion.

### The concentration of root *t*Z-type cytokinins is impaired in the incompatible graft in the presence of fruit

To investigate the influence of the graft combination and of fruiting on the trafficking of cytokinins, scion sap (above the graft junction) and rootstock sap (below the graft junction) were subjected to cytokinin profiling. Regardless of the treatment, total *t*Z-type cytokinin level was significantly higher in the scion sap of Ki/TZ than Ki/Ki and Ki/53 but in the presence of fruit, *t*Z-type cytokinins decreased in Ki/r53 and increased in Ki/Ki (Supplementary Fig. 3A). In the presence of fruit, the content of *c*Z-type cytokinins increased in the scion sap in Ki/r53, but no changes were observed in Ki/Ki and Ki/TZ (Supplementary Fig. 3B). In contrast, in the presence of fruit, the content of iP-type cytokinins in the scion sap was similar for all three combinations (Supplementary Fig. 3C).

In the presence of fruit, the content of *t*Z-type cytokinins decreased in the rootstock sap of Ki/TZ and Ki/r53 but remained unchanged in Ki/Ki (Supplementary Fig. 3D), while the content of *c*Z-type cytokinins was significantly higher in Ki/r53 than in Ki/TZ and Ki/Ki (Supplementary Fig. 2E). In contrast, in the presence of fruit, the levels of iP-type cytokinins in the rootstock sap were similar for all graft combinations (Supplementary Fig. 3F). These results show that the fruiting process in the control Ki/Ki increases in scion sap *t*Z-type cytokinins content, but not *c*Z-type cytokinins content (Supplementary Figs. 3A, B). On the other hand, fruiting did not affect *t*Z-type or *c*Z-type cytokinins levels in the Ki/Ki rootstock sap (Supplementary Figs. 3D, E). Furthermore, our results also showed high levels of *c*Z-type cytokinins (leaf synthesized cytokinins) in the rootstock sap of the incompatible combination and similar levels of iP-type cytokinins in the rootstock sap of all three grafts combinations, thus refuting the notion of a blockage at the graft junction.

**Figure 3:**
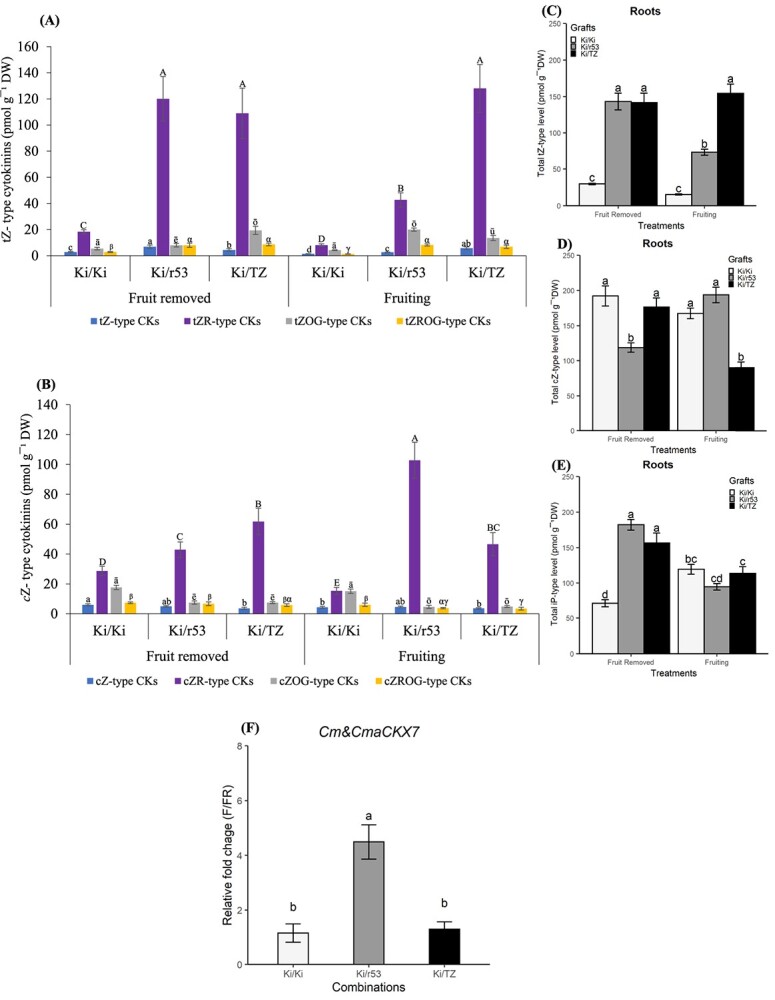
**Profiles of cytokinins and relative expression of cytokinin oxidase/dehydrogenase 7 (*CKX7*) in roots collected during fruit ripening from Ki/Ki, Ki/r53, and Ki/TZ grown under two treatments, i.e. fruiting and fruit removed.** (A) Root *t*Z-type cytokinins; (B) root *cZ-*type cytokinins; (C) total root type *t*Z-type cytokinins; (D) total root *c*Z-type cytokinins; (E) total root iP-type cytokinins; and (F) root *Cm&CmaCKX7* expression. The fold change was calculated using the expression level of fruiting plants vs. fruit-removed plants, and it is represented by the mean value and standard error of the relative levels of expression of the target gene normalized to *β-actin* expression. Data are means ± se (*n* = 4 biological replicates). Purple columns with different capital letters indicate differences in the levels of *tZR-*type cytokinins; blue columns with different lowercase letters denote differences between *tZ-*type cytokinins; gray columns with different lowercase letters with a bar above (ā, ē, or ō) show differences in the *tZOR-*type cytokinins; and yellow columns with different Greek letters indicate differences in *tZOR-*type cytokinins. Columns with different letters indicate significant differences between graft combinations and treatment (fruit removed and fruiting). Two-way ANOVA *p* < 0.05, as determined by Tukey–Kramer HSD.

To elucidate how the levels of the root-synthesized *t*Z-type and *c*Z-type cytokinins are affected by scion-rootstock (in)compatibility in fruiting plants, profiling of root cytokinins was performed. The results revealed significant differences between the different graft combinations and treatments in both *t*Z-type cytokinins [namely, *t*ZR, *trans*-zeatin-O-glucoside (*t*ZOG), and *trans*-zeatin-O-glucoside riboside (*t*ZROG) cytokinins] and *c*Z-type cytokinins [namely, *c*ZR, *cis*-zeatin-O-glucoside (*c*ZOG), and *cis*-zeatin-O-glucoside riboside (*c*ZROG) cytokinins] (Figs. 3A, B). The levels of *t*ZR-type cytokinins were higher in the roots of Ki/TZ and Ki/r53 than in Ki/Ki in the absence of fruit, but in the presence of fruit*,* levels decreased in Ki/Ki and Ki/r53 but remained unchanged in Ki/TZ (Fig. 3A). In the presence of fruit, the level of *t*ZOG in the roots increased in Ki/r53, decreased in Ki/TZ, and remained unchanged in Ki/Ki (Fig. 3A). In the presence of fruit, the level of *t*ZROG in the roots decreased in Ki/Ki but remained unchanged in Ki/TZ and Ki/r53 (Fig. 3A).

In the presence of fruit vs. fruit removal, root levels of *c*ZR-type cytokinins were higher in Ki/r53, decreased in Ki/Ki, and were similar in Ki/TZ (Fig. 3B); the level of *c*ZOG decreased in both heterograft combinations but remained similar in Ki/Ki (Fig. 3B), and the level of *c*ZROG decreased in Ki/TZ and remained similar in Ki/Ki and Ki/r53 (Fig. 3B). However, in the absence of fruit, the level of root *c*ZR-type cytokinins was significantly higher in Ki/TZ than in Ki/r53 and Ki/Ki (Fig. 3B).

When the findings were analyzed in terms of total *t*Z-type cytokinins and total *c*Z-type cytokinins, major differences emerged between the different graft combinations. In particular, in the presence of fruit, a decrease of total *t*Z-type cytokinins and an increase in total *c*Z-type cytokinins were observed in the roots of the incompatible Ki/r53 plants (Figs. 3C, D). In contrast, in the compatible Ki/TZ plants, similar levels of total *t*Z-type cytokinins were observed in both treatments (fruit removed and fruiting) in concert with decreased levels of total *cZ*-type cytokinins during fruiting (Figs. 3C, D). In the self-grafted Ki/Ki, total *t*Z-type and *c*Z-type cytokinins levels remained unchanged, regardless of the treatment (Figs. 3C, D). Importantly, in the roots of self-grafted Ki/Ki plants, the levels of total *c*Z-type cytokinins were ~ 6 times higher than those of total *t*Z-type cytokinins (Figs. 3C, D), in keeping with previous findings that pumpkin synthesizes more *t*Z-type cytokinins than melon (Fig. 3C). In the presence of fruit, decreased levels of root iP-type cytokinins were found in both heterograft combinations, while in the self-grafted combination, an increase was observed (Fig. 3E). The above-described results show that the synthesis and accumulation of cytokinins in the roots vary with scion-rootstock combination, level of compatibility, and treatment.

A possible explanation for these findings was thought to lie in the role of cytokinin oxidase/dehydrogenase (*CKX*) in catalyzing the irreversible degradation of cytokinins. Therefore, the levels of *CKX7*, a cytosolic *CKX* protein, were assayed to determine whether fruiting and incompatibility induce changes in *CmaCKX7* expression. Importantly, *CmaCKX7* relative expression was ~3.5 times higher in the roots of the incompatible Ki/r53 plants than in the compatible Ki/TZ and self-grafted Ki/Ki plants (*CmCKX7*) (Fig. 3F).

### Root IAA homeostasis is perturbed in the incompatible graft combination in the presence of fruit

IAA content was determined in scion and rootstock sap to investigate the influence of the graft combination and of fruiting on the trafficking of IAA. IAA profiling analysis revealed that the fruiting process led to significantly decreased IAA levels in the scion sap and increased levels in the rootstock sap of Ki/r53 and that fruiting increased the IAA concentration in the Ki/TZ scion sap and rootstock sap in a similar manner (Supplementary Figs. 4A, B). The data also showed normal IAA flow across the graft junction in Ki/r53 and suggested that different IAA levels in scion sap and the rootstock sap of this combination probably derived from the low compatibility between the scion and the rootstock, which was exacerbated by the fruiting process.

**Figure 4:**
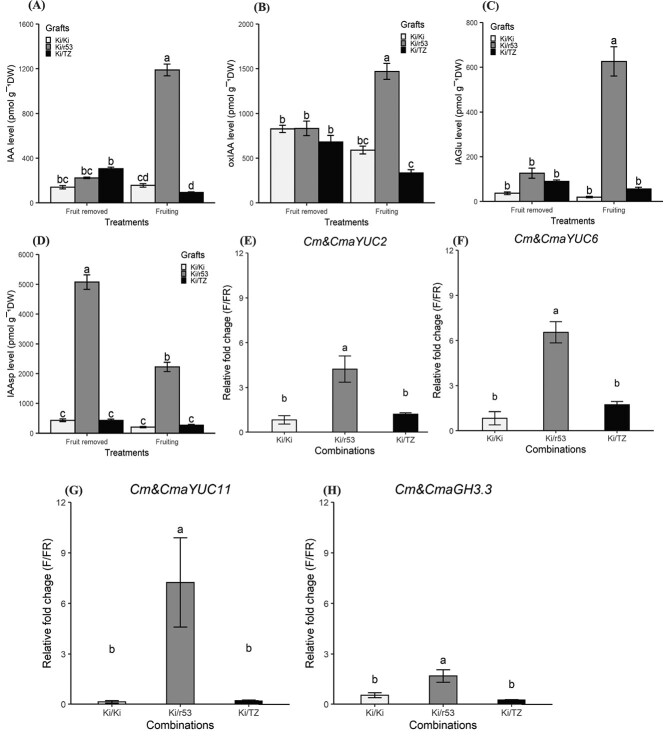
**Root auxin (IAA) profile and relative expression levels of *YUCCA* (*YUC*) and *Gretchen Hagen3* (*GH3.3*) genes in the roots of Ki/Ki, Ki/r53, and Ki/TZ plants.** (A) IAA content; (B) 2-oxoindole-3-acetic acid (oxIAA) content; (C) indole-3-acetic glutamate (IAGlu) content; (D) indole-3-acetic aspartate (IAAsp) content; (E) *Cm&CmaYUC2* expression; (F) *Cm&CmaYUC6* expression; (G) *Cm&CmaYUC11* expression; and (H) *Cm&CmaGH3.3* expression. Data are means ± se (*n* = 4 biological replicates). Bars with different letters indicate significant differences between graft combinations and treatments (fruiting and fruit removed). Two-way ANOVA *p* < 0.05, as determined by Tukey–Kramer HSD.

Based on our premise that graft incompatibility induces local IAA accumulation in the roots during fruit ripening, the levels of IAA in leaves and roots were studied for the two treatments, i.e. fruiting and fruit removed. For all three graft combinations, no statistically significant differences were observed in leaf IAA levels between the two treatments (Fig. 1B). In contrast, in the presence of fruit, IAA content in the roots was ~7 times higher in the incompatible Ki/r53 graft than in the compatible Ki/TZ and self-grafted Ki/Ki combinations (Fig. 4A). In seeking an explanation for the higher IAA content in the roots of the incompatible plants (namely, enhanced IAA synthesis or impaired IAA oxidation and/or conjugation pathways), we determined oxIAA, IAGlu, and IAAsp contents in the roots of the three combinations. In the presence of fruit (vs. fruit removed), similar patterns were observed for oxIAA and IAGlu contents in the roots, i.e. the levels were ~ 2 and ~ 7 times higher, respectively, in the incompatible Ki/r53 plants than in the compatible Ki/TZ plants (Figs. 4B, C). Again, no statistically significant differences were observed in the self-grafted Ki/Ki plants (Figs. 4B, C). In contrast, the IAAsp content was ~6 and ~ 2 times higher in the absence and presence of the fruit, respectively, in the roots of the incompatible Ki/r53 plants than in the self-grafted Ki/Ki plants and the compatible Ki/TZ grafts (Fig. 3D). Notably, the levels of IAGlu and IAAsp were similar in the self-grafted Ki/Ki and the compatible Ki/TZ plants, regardless of the treatment (Figs. 4C, D). These results strongly support the notion that root IAA homeostasis is altered in the incompatible graft combination in the presence of fruit.

Excessive local IAA accumulation in the roots – as a result of translocation from the scion to the rootstock or of IAA synthesis in the roots – may trigger graft collapse in incompatible combinations in the presence of fruit. Therefore, as a complementary route to investigating the dramatically enhanced IAA level in the roots of the incompatible graft combination in the presence of fruit, we performed real-time quantitative polymerase chain reaction (RT-qPCR)-based transcriptomic analysis. The fold change (fruiting vs. fruit removed) in the expression of *CmYUC2* and of *CmYUC6* was ~3.5, and ~ 2 times higher in the leaves of Ki/r53 compared with Ki/TZ leaves (Supplementary Figs. 4C, D) and ~ 2 and ~ 3.5 times higher compared with Ki/Ki leaves (Supplementary Figs. 4C, D). No significant differences were found in *CmYUC11* expression between the graft combinations (Supplementary Fig. 4E).

Importantly, *CmaYUC2*, *CmaYUC6* and *CmaYUC11* were ~ 4, ~6 and ~ 6.5 times higher in the roots of the incompatible Ki/r53 combination than in those of the compatible Ki/TZ combination (Figs. 4E-G). The fold change (fruiting vs. fruit removed) in the expression of *CmYUC2*, *CmYUC6* and *CmYUC11* in the self-grafted Ki/Ki roots was similar to that in the compatible Ki/TZ roots and lower than that in the incompatible Ki/r53 roots (Figs. 4E-G).

In the presence of fruit, the fold change in *CmaGH3.3* expression was significantly higher (~1.6) in the roots of the incompatible Ki/r53 plants than in those of the compatible Ki/TZ and self-grafted Ki/Ki plants (Fig. 4H). These results suggest that IAA accumulation in the roots is associated with higher expression of *YUC* genes.

### Root IAA/cytokinins ratio differs between the compatible and incompatible graft combinations in the presence of fruit

**Figure 5:**
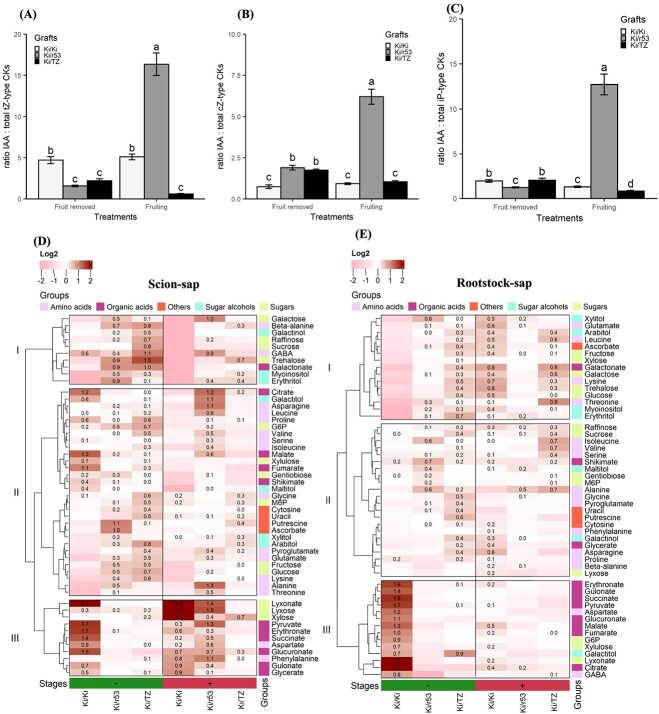
**Auxin:cytokinins ratio and trafficking of photoassimilates in Ki/Ki, Ki/r53, and Ki/TZ under two treatments, i.e. fruiting and fruit removed.** (A) Root auxin:*t*Z cytokinin ratio; (B) root auxin:*c*Z cytokinin ratio; and (C) root auxin:iP cytokinin ratio; (D) heat map showing the hierarchical clustering of the metabolite log_2_ values in the scion sap; and (E) heat map showing the hierarchical clustering of the metabolite log_2_ values in the rootstock sap. Bars with different letters indicate significant differences between graft combinations and treatments (fruit removed and fruiting). Two-way ANOVA *p* < 0.05, as determined by Tukey–Kramer HSD. In the heat maps dark red indicates a high relative abundance of metabolites, and light pink indicates a low relative abundance.

Further, we posited that the high level of IAA in the roots of the incompatible combination disrupts the homeostasis of both IAA and cytokinins. Therefore, we calculated the ratio between the root IAA content and the total *t*Z-, *c*Z-, and iP-type cytokinins contents to test this notion. The results indicate that the dramatic accumulation of IAA in the roots of the incompatible Ki/r53 fruiting plants (Fig. 4A) was reflected in IAA/total *t*Z-type cytokinins ratios that were ~ 16 times and ~ 10 times higher in Ki/r53 plants than in the compatible Ki/TZ and self-grafted Ki/Ki plants, respectively (Fig. 5A). In addition, due to the low level of total *t*Z-type cytokinins in the roots of self-grafted Ki/Ki plants (regardless of the treatment; Fig. 3I), the IAA/total *t*Z-type cytokinins ratio was ~4 times higher in the self-grafted Ki/Ki plants than in the compatible Ki/TZ plants (Fig. 5A). Finally, in the presence of fruit, the IAA/total *c*Z-type cytokinins and IAA/total iP-type cytokinins ratios of the incompatible Ki/r53 combination were ~ 12 and ~ 10 times higher than the ratios for the compatible Ki/TZ and the self-grafted Ki/Ki combinations (Figs. 5B, C).

We broadened this part of the investigation to cover the trafficking of photoassimilates from the scion to the rootstock. An analysis of sap collected at fruiting stage (~ 140 DAG) showed that in fruiting plants (colored red and marked with a plus sign in Fig. 5D), the transport rate of photoassimilates decreased mainly in Ki/r53, leading to their accumulation in the scion sap, while a reduction in the rootstock sap was evident (Fig. 5D, E) while in Ki/Ki and Ki/TZ only a few metabolites were accumulated in the scion sap (Fig. 5D). Importantly, in fruiting plants, the most significant metabolites accumulating in the Ki/r53 scion sap were γ-aminobutyric acid (GABA), citrate, galactitol, asparagine, pyruvate, and phenylalanine (Fig. 5D), resulting in their reduction in rootstock sap (Fig. 5E). Furthermore, in the Ki/Ki scion sap, the accumulation of lyxonate, lyxose, and xylose were slightly high in fruiting plants vs. fruit-removed plants (Fig. 5D cluster III). In contrast, in the scion sap metabolites in Ki/TZ the accumulation rate was lower in fruiting plants vs. fruit-removed plants (Fig. 5D cluster III).

In Ki/Ki rootstock sap, when fruits were removed (colored green and marked with a minus sign in Fig. 5E), the relative abundance of metabolites in cluster I was high vs. fruiting plants but the relative abundance of metabolites in cluster II and III was lower vs. fruiting plants (Fig. 5E). On the other hand, in Ki/TZ rootstock, metabolites accumulation was almost similar in fruiting and fruit removed plants (Figs. 5F). Taken together, these results suggest that a disruption of the IAA–cytokinins balance in the incompatible graft combination (Ki/r53) during fruiting is closely associated with a reduction of photoassimilates in the rootstock.

### H₂O₂ and MDA contents are enhanced and antioxidant enzyme activity is reduced in the roots of the incompatible graft in the presence of fruit

**Figure 6:**
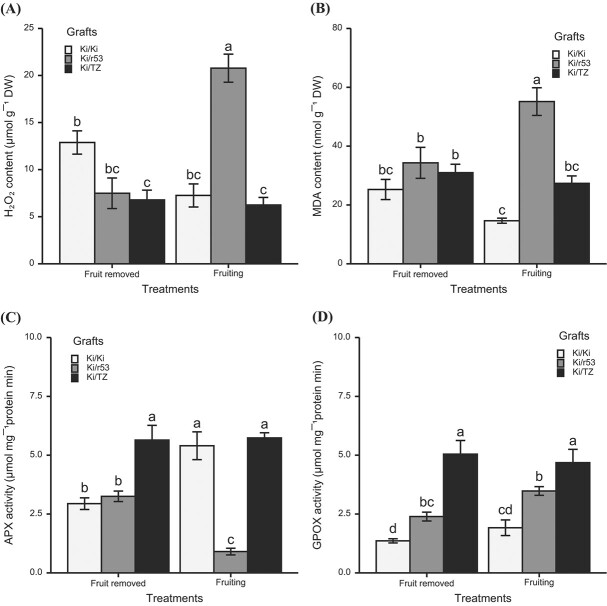
**H**_**2**_ **O**_**2**_ **and malondialdehyde (MDA) levels and ascorbate peroxidase (APX) and guaiacol peroxidase (GPOX) activity in Ki/Ki, Ki/r53 and Ki/TZ under two treatments, i.e. fruiting and fruit removed.** (A) Root H₂O₂ content; (B) root MDA content; (C) root APX activity; and (D) root GPOX activity. Data are means ± se (*n* = 4 biological replicates). Bars with different letters indicate significant differences between graft combinations and treatments (fruit removed and fruiting). Two-way ANOVA *p* < 0.05, as determined by Tukey–Kramer HSD.

To provide support for our premise that high IAA levels in the roots of the incompatible graft combination in the presence of fruit induce oxidative stress, we investigated whether high IAA accumulation triggers H₂O₂ accumulation, which might, in turn, lead to MDA accumulation (H₂O₂ and MDA are both indicators of oxidative stress). In the presence of fruit, the roots of the incompatible Ki/r53 plants did indeed contain a higher level of H₂O₂ (Fig. 6A). However, no differences were observed in the levels of H₂O₂ in the compatible Ki/TZ and self-grafted Ki/Ki roots in plants, with or without fruit (Fig. 6A). MDA content was significantly higher in the roots of the incompatible Ki/r53 fruiting plants and similar in the compatible Ki/TZ and self-grafted Ki/Ki fruiting plants vs. plants whose fruit had been removed (Fig. 6B).

Plants deal with oxidative stress primarily via an endogenous defensive mechanism comprising different enzymes, including ascorbate peroxidase (APX) and guaiacol peroxidase (GPOX), among others [31]. Thus, as an additional measure of oxidative stress, we also studied the activity of APX and GPOX. In the presence of fruit, a significant decrease in APX activity was observed only in the incompatible Ki/r53 roots, while APX activity was enhanced in the self-grafted Ki/Ki roots and remained unchanged in the compatible Ki/TZ roots (Fig. 6C). In contrast, GPOX activity in the roots was statistically similar in all the graft combinations in both treatments, i.e. with and without fruit (Fig. 6D).

### Elevated root concentrations of IAA and *cZ*-type cytokinins but a lower concentration of *tZ*-type cytokinins and a scion accumulation of metabolites characterized collapsed Ki/r53 plants

To further investigate the accumulation of root IAA and cytokinins in collapsed Ki/r53 plants (as manifested by wilting), IAA, *tZ*-type cytokinins, and *cZ*-type cytokinins were quantified in roots collected from fruit-removed, fruiting, and fruiting collapsed plants. Excessive IAA accumulation in the roots was observed in the collapsed plants vs. the fruiting and fruit-removed plants (Supplementary Fig. 5A). In contrast, the concentration of root *tZ*-type cytokinins declined as the collapse began (Supplementary Fig. 5B), and that of rootstock *cZ*-type cytokinins gradually increased (Supplementary Fig. 5C).

In addition, differences in local accumulation (scion, graft junction, and rootstock) of the stem metabolites in fruit-removed, fruiting, and collapsed plants were examined. The stem metabolites data set revealed that the main metabolites accumulating locally in the scion stem of fruiting collapsed plants were amino acids, including glycine, valine, and lysine (Supplementary Fig. 5D; cluster II), and alanine, isoleucine, proline, and GABA (Supplementary Fig. 5D; cluster III). In addition, concentrations of erythritol (Supplementary Fig. 5D; cluster II), ascorbate and trehalose (Supplementary Fig. 5D; cluster III), and several other metabolites (Supplementary Fig. 5D; cluster II, III) were higher in the scion of fruit-removed plants than in fruiting and collapsed plants. In addition, high relative abundances of arabinose, xylose, lyxose, and lyxonate were found in the collapsed plants’ rootstock stem (Supplementary Fig. 5D; cluster I). At the graft junction, metabolites in clusters II and III were slightly higher in the fruit removed than fruiting and collapsed plants; however, no clear trend in terms of accumulation of metabolites was observed (Supplementary Fig. 5D; cluster I). Notably, the relative abundances of 23 metabolites were low in the scion stem but high in the rootstock stem in fruit-removed, fruiting, and fruiting collapsed plants (Supplementary Fig. 5D; cluster I) and vice versa (Supplementary Fig. 5D; cluster III). Therefore, the high relative accumulation rate of stress response metabolites such as GABA, proline, trehalose, isoleucine, and alanine in the scion stem of fruit-collapsed plants might indicate a response to oxidative stress.

## Discussion

Interspecific grafting often enhances plant vigor [32], but some combinations, such as melon grafted onto pumpkin rootstock, can manifest incompatibility months after grafting [6, 8, 33]. This study of compatible, incompatible, and self-grafted combinations showed premature rootstock senescence in the incompatible graft regardless of the treatment (fruiting or fruit removal). The three grafted combinations showed minor or no changes in scion-expressed in leaf metabolites, total leaf chlorophyll, and IAA content but significant differences in the concentrations of cytokinins between self-grafted and heterografted plants in the presence of fruit. Moreover, the incompatibility did not impose a major obstacle to plant growth, as shown by the slight variation in the constitution of the leaf metabolome and the accumulation of IAA and cytokinins in the leaves during the vegetative, flowering, and fruit ripening stages—even the incompatible Ki/r53 plants until the collapse began. These findings are in keeping with those of a previous study showing that the rootstock declined in the early collapse of melon-pumpkin grafts while the scion remained healthy [9]. In that study, the oxidative stress at the graft union was attributed to the accumulation of H_2_O_2_ and the low activity of enzymes related to H_2_O_2_ degradation, leading to the collapse of the incompatible melon-pumpkin combination at 24 DAG [9]. It was proposed that the oxidative stress was probably triggered by hormonal signaling (such as coordinating nutrition deficiencies and enhancing the acropetal transport of cytokinins via a decrease in IAA flow to the roots) from the scion to the rootstock [11]. It was also proposed that the above-threshold concentrations of H_2_O_2_ probably affect rootstock function by impairing DNA [34], thereby eliciting premature senescence. However, none of these suppositions could explain the reason for graft collapse; instead, they highlighted the symptoms of graft incompatibility, namely, chlorophyll degradation and increased H_2_O_2_ content below graft junction and the early senescence of the rootstock.

The fruiting process induces the translocation of root carbohydrates for fruit development[35]^,^ which might affect the hormonal status. For example, a recent study showed that the rootstock sap contained a higher level of *t*Z-type cytokinins during fruit ripening, and the scion showed almost no accumulation of photoassimilates in a self-grafted pumpkin (r53/r53) than the incompatible melon-pumpkin graft combination Ki/r53 [12]. Similarly, a study on grafted watermelon suggested that a reduction in cytokinins (mainly *t*Z-type cytokinins) in the xylem sap during fruit ripening could be associated with a reduction in the transport of photoassimilates from the scion to rootstock [36]. In the current study, a fruit removal experiment was conducted to examine possible changes in hormone accumulation and trafficking of photoassimilates induced by fruiting. Our findings demonstrated that in the incompatible Ki/r53 combination fruiting triggers a reduction in the rate of translocation of photoassimilates or induces the translocation of photoassimilates from the root to the fruit, a notion supported by the accumulation of a significant number of metabolites (mainly amino acids and sugars) in the scion sap. The accumulation of metabolites in the scion was accompanied by a decrease in *tZ*-type cytokinins and enhanced accumulation of *cZ*-type cytokinins in the scion and rootstock sap and the roots. Conversely, in fruit-removed plants, higher levels of *tZ*-type cytokinins were observed in the roots of the two heterograft combinations than in the roots of the self-grafted plants. In addition, an analysis of IAA and *t*Z-type and *c*Z-type cytokinins in the roots of the incompatible Ki/r53 plants (fruit removed, fruiting, and fruiting collapsed) showed that accumulation of *t*Z-type cytokinins decreased threefold in collapsed plants vs. fruit-removed plants, and the accumulation of *c*Z-type cytokinins and IAA increased ~threefold in collapsed plants vs. fruit-removed plants. It is possible that the increase in *c*Z-type cytokinins in the roots of fruiting Ki/r53 plants could be due to the downregulation of the expression of *CKX7*, which preferentially degrades *c*Z-type cytokinins [37]. Surprisingly, however, we found upregulation of *CmaCKX7* in the roots of Ki/r53, implying that *CKX7* might act via reducing the accumulation of *tZ*-type cytokinins. This possibility is refuted by a study performed on *Arabidopsis* seedlings, which showed that *ATCKX7* preferentially degrades *c*Z-type cytokinins [37]. However, in line with our findings, a different study – one using the *Arabidopsis* mutants, the *stk* (MADS-box transcription factor SEEDSTICK, which directly regulates the expression of *CKX7*) mutant and two *ckx7* (*CKX7*, which degrades cytokinins) mutants – provided evidence of increased levels of *tZ*-type cytokinins during the fruit elongation stage [38]. Taken together with our results, these data suggest that *CKX7* might have different substrate affinities toward *tZ*-type cytokinins at different plant physiological stages. This premise, however, requires further investigation.

IAA influences almost every part of plant growth and development by modulating cell expansion and elongation [39]; thus, IAA overaccumulation in Ki/r53 roots of fruiting plants suggested that the photoassimilate reduction in the root might enhance roots’ IAA synthesis or/and its transport to promote root growth. Support for this notion may be drawn from the high expression of *CmaYUC2*, *CmaYUC6*, and *CmaYUC11* (which are involved in IAA synthesis) in the roots and high high *CmYUC2* and *CmYUC6* in leaves only in Ki/r53 but similar leaf IAA concentrations in all three graft combinations. Moreover, high IAA concentrations in the roots of fruiting collapsed plants also support our premise (Supplementary Fig. 5A). In addition, in the presence of fruit (vs. fruit removed), the lower level of IAA in the scion sap and the high level in the rootstock sap of Ki/r53 strongly suggested local IAA accumulation, as in Ki/Ki and Ki/TZ both scion and rootstock sap contained similar levels of IAA. Therefore, in contradiction to the suggestion of Aloni et al. [10] that threshold levels of root auxin (produced in the scion and translocated to the roots) trigger the root degradation, we posit that IAA accumulation in the roots of the incompatible combination does not derive solely from auxin transport, but rather, it is due both to IAA transport from the scion to the roots and to root-produced IAA.

In light of the above, we posited that the IAA overproduction observed in the roots of the incompatible plants could result from an impairment in the IAA regulation mechanism, and we, therefore, extended the study to the two IAA conjugation products, IAGlu and IAAsp, and the product of IAA oxidation, oxIAA. Our results showed that root oxIAA, IAAsp and IAGlu contents increased in the incompatible graft in the presence of fruit (Figs. 4C, D). In line with our results, accumulation of IAAsp and IAGlu were observed in *Arabidopsis* after exogenous treatment with high levels of IAA, thus negating the possibility that impairment of the IAA regulation pathway was responsible for the enhancement of IAA accumulation in the roots of the incompatible combination. In addition, qRT-PCR–based transcriptomic analysis revealed high expression of *CmaGH3.3* in the roots of Ki/r53 (Fig. 5C). It has been shown that high expression of *GH3.3*, promoted by high levels of IAA, quickly regulates IAA to the basal level by conjugating it to amino acids to form compounds such as IAGlu and IAAsp [25]. In light of the above, it is evident that the root IAA overaccumulation observed in our study was not due to the impairment of the IAA regulation pathway. Overall, our results indicate that, at high IAA levels, the most significant IAA degradation pathways [26] remained functional in the roots of the incompatible combination. Nonetheless, IAA levels in the root of Ki/r53 were high, indicating that IAA homeostasis is regulated in a complex manner.

Impairment of IAA homeostasis can lead to a hormonal imbalance, and, indeed, Aloni et al. [11] suggested that an IAA–cytokinin imbalance might trigger the collapse of grafted melon plants. In support of such a notion, this study shows that fruiting imposed a decline in rootstock photoassimilates, which apparently affects the regulation of IAA and cytokinins in the roots, with the regulation being intimately dependent on the graft combination. For example, about 90% of the incompatible melon-pumpkin grafted plants collapsed during fruit ripening, a process that occurred in parallel with an alteration in the IAA:cytokinins ratio and the accumulation of photoassimilates in the scion. In keeping with these findings, a study in grafted peach showed that reducing IAA transport from the rootstock to the scion via the xylem increased root synthesis of *t*ZR cytokinins in the scion [40], highlighting the close interaction between IAA and cytokinins. This intimate association led us to the idea that some graft combinations may enhance either *t*ZR synthesis (leading, in turn, to lower IAA levels in the roots) or IAA synthesis (leading to a reduction in *t*Z-type cytokinins in the rootstock). This notion is supported by similar levels of IAA and *t*Z-type cytokinins in the roots of compatible and incompatible graft combinations in the absence of fruit and likewise by a similar trend of photoassimilates accumulation in the scion and rootstock sap. Despite the different mechanisms involved in the biosynthesis, accumulation, and translocation of cytokinins and IAA, it is clear that in the incompatible combination, the fruiting processes and the incompatibility itself both modulated the accumulation of *t*Z-type and *c*Z-type cytokinins and IAA in the roots and the translocation or/and accumulation of photoassimilates, suggesting that root cytokinins and IAA concentrations are regulated by a source/sink relationship.

Cell damage under stress conditions depends on accumulation of ROS, such as H_2_O_2_ [31, 41]; for example, Aloni et al. [9] showed that ROS increased in the graft zone in incompatible graft combinations in young plants. In parallel, it has been posited that high levels of IAA could induce ROS production [42, 43], with above basal concentrations of ROS, such as H_2_O_2,_ and MDA, causing oxidative stress, which in most cases leads to senescence [44, 45]. Moreover, studies in melon-pumpkin grafts have suggested that the transport of IAA in high concentrations from the scion to susceptible rootstock will trigger ROS formation in the roots, eliciting plant collapse [10, 11]. The current study found that increases in endogenous IAA levels in the roots were synchronized with elevations in H_2_O_2_ and MDA levels in the presence of fruit. However, we did not find any differences between self-grafted and heterograft combinations in the absence of fruit—the condition that was used as a control to measure the effect of fruiting on graft collapse. Further, we demonstrated that APX activity, which plays a pivotal role in preventing H_2_O_2_ toxicity [46] by ROS scavenging, was lower in the roots of the incompatible combination than in those of the self-grafted and compatible combinations. The question of whether IAA accumulation directly causes H_2_O_2_ production and reduced APX activity is beyond the scope of this work. However, it was previously shown that exogenous application of IAA increased the concentration of H_2_O_2_ in the root tips of wild-type tomato compared with the *dgt* (auxin-resistant) mutant [43]. In addition, work on *Arabidopsis* mutants with impaired ROS scavenging ability exhibited ROS accumulation and consequent triggering of oxIAA formation [42], thus supporting our hypothesis that IAA overproduction could lead to the accumulation of H_2_O_2_ and ultimately to MDA production.

Taken together, this study provided evidence that graft incompatibility expressed during fruiting encompasses a complex process that induces transport changes and/or accumulation of hormones and photoassimilates. A deficiency of photoassimilates sensed in the root system – where hormones serve as essential signaling components in terms of developmental reprogramming – will elicit changes in the root-to-shoot and/or shoot-to-root signals, delivered through the xylem and phloem, respectively, and involving *c*Z-type cytokinins, *t*Z-type cytokinins, and IAA. IAA–cytokinine imbalance possibly induced increases of H_2_O_2_ and MDA and decreased oxidative stress enzymes’ activity, ultimately leading to graft collapse.

## Materials and methods

### Plant material

The melon (*Cucumis melon* L.) cultivar “Kiran” (designated Ki) was used as the scion. Two interspecific pumpkin hybrids (*C. maxima* Duch. × *C. moschata* Duch.) were used as the rootstock, a commercially available compatible hybrid “TZ-148” (designated TZ), and “53 006” (designated r53), which was experimentally characterized by low compatibility with Ki. In addition, Ki was self-grafted and used as the control. Grafting was performed manually at Hashtil Nurseries Co. (Ashkelon, Israel), as described in Camalle et al. [12].

### Growth conditions and sample collection

Two independent experiments were conducted in a greenhouse on the Sede Boqer Campus of Ben-Gurion University of the Negev from May 2018 to September 2018 and from May 2019 to September 2019. In both experiments, the plants were transplanted at 40 DAG into 10-L pots filled with sand. Plants of each combination, i.e. Ki/TZ, Ki/r53, and the self-grafted Ki/Ki, were randomly distributed in four blocks, with five plants per block. Plants were grown at 30/20°C, with daylight dropping from about 14 h in May to about 12 h in September. The plants were fertigated twice a day for the first two weeks after transplant with 0.015% (N-P-K); thereafter, they were fertilized with 0.03% N-P-K + micronutrients (Ca^2+^ and Mg^2+^) until the end of the experiment. Daily irrigation was split into two cycles, given in a total volume of 1.5 L per day. To determine whether fruiting influences the hormonal balance, at 10 days after anthesis (DAA), the plants of each of the three grafting combinations were divided into two groups, one (designated fruiting) in which 100% of the fruit would be allowed to develop on the plants and the other (designated fruit removed) in which all the fruit would be removed manually approximately 88 DAG or 43 days after transplant (Supplementary Fig. 1A). It should be noted that, for the 2019 experiment, samples were collected from fruit removed, fruiting, and collapsed plants (fruiting plants that collapsed during the fruit ripening stage); the data reported pertain to incompatible combination, unless otherwise specified.

For the profiling of hormones (cytokinins and IAA) and metabolites, fully developed youngest leaves (Supplementary Fig. 1B) were sampled from the plants at ~2 weeks after the start of the experiment (vegetative stage ~54 DAG), at the flowering stage (~78 DAG or ~ 3.5 weeks of first sampling), and at the ripening stage (~130 DAG) (Supplementary Fig. 1C). Roots, sap from scion and rootstock, and stem (e.g. scion, grafted junction, and rootstock) samples were taken at the fruit ripening stage (130–140 DAG), i.e. when the fruit turned yellow (Supplementary Fig. 1C), and the collapse of the incompatible Ki/r53 combination was evident. Samples were collected as follow: Approximately 1 g of third-node leaves were collected from the canopy of each combination; stem samples were cut out 2 cm above and below the graft; roots were collected, washed and dried with paper toweling (Supplementary Fig. 1B); and sap exudate (four biological replicates per combination) was collected from the scion and rootstock, as described in Camalle et al. [12]. Leaf, stem, root, and sap samples were immediately frozen in liquid nitrogen and stored at −80°C prior to lyophilization. The lyophilized sap was ground under liquid nitrogen in a TissueLyzer (RetschGmbh & Co. KG, Germany) containing pre-chilled holders and beads. Powered material was stored at −80°C until analysis. Four biological replicates pooled from five plants were used for all analyses; the biological replicates were analyzed with three technical replicates.

### Rootstock-stem total chlorophyll extraction and quantification

Visual symptoms of early rootstock senescence in the incompatible combination were previously reported by Camalle et al. [12]. Here, we complemented the previous findings by evaluating rootstock-stem total chlorophyll content, since chlorophyll degradation is one of the symptoms of senescence [47]. Chlorophyll was extracted and quantified as described in [48]. The values obtained for chlorophyll a and b were used to calculate the total chlorophyll content, as described in [49]. The stem total chlorophyll content was expressed as mg total chlorophyll g^−1^ dry weight (DW).

### Leaf, root and sap hormone profiling by LC–MS/MS

LC–MS/MS was used to determine endogenous levels of cytokinins, free IAA, the IAA catabolite, oxIAA, and the amino acid conjugates, IAGlu and IAAsp [22, 50]. The extracts were purified on an Oasis MCX column (30 mg/mL, Waters) [51]. Phytohormones were determined using an ultra-high performance liquid chromatography-electrospray tandem mass spectrometry system (Acquity UPLC I-Class System coupled to a Xevo TQ-S MS, all from Waters) using stable isotope-labeled internal standards as a reference [52].

### Leaves, sap, and scion- and rootstock-stem metabolomic profiling by GC–MS/MS

Metabolite extraction was performed as described in Camalle et al. [12].

### Determination of the endogenous H_2_O_2_ concentration in stems and roots

For determination of H_2_O_2_ accumulation, 10-mg samples of lyophilized stems or roots were homogenized with 1 mL of 1 M perchloric acid (Sigma-Aldrich, CAS: 67–56-1) containing 5% polyvinylpyrrolidone. The homogenate was centrifuged at 10000 rpm for 10 min at 4°C. Thereafter, the supernatant was diluted ×2, neutralized with 5 M potassium carbonate in the presence of 50 μL of 300 mM potassium buffer (P-buffer), and centrifuged at 10000 rpm for 1 min. The centrifugate was supplemented with 50 mM Tris–HCl buffer, pH 6.5, 8.5 mM 4-aminoantipyrine, 3.4 mM sodium 3,5-dichloro-2-hydroxybenzenesulfonate, and 45 U/mL horseradish peroxidase. H_2_O_2_ content was determined spectrophotometrically in 200-μL samples at 515 nm (Epoch Microplate Spectrophotometer, BioTek) with Gen5 2.05 software. Spectrophotometer readings were calibrated against a standard curve for known concentrations of 3% H_2_O_2_ (Sigma-Aldrich St. Louis, MO, USA) diluted in 50 mM P-buffer (pH 7) to concentrations ranging between 0 and 200 nM.

### Determination of endogenous MDA concentration in roots

MDA concentration in the roots was determined, as described previously [53], for all graft combinations in the presence and absence of fruit. MDA content was determined spectrophotometrically in 200 μL of supernatant at 440, 532, and 600 nm. MDA content was expressed as nmol g^−1^ DW.

### Determination of ascorbate peroxidase and guaiacol peroxidase activities in roots

To evaluate possible differences in the root enzymatic balance in the presence and absence of fruit, root APX and GPOX activities were determined for all graft combinations, as described previously [54]. APX and GPOX were determined spectrophotometrically in 200 μL of supernatant at 290 and 470 nm, respectively, with an Epoch Microplate Spectrophotometer, BioTek with Gen5 2.05 software.

### RNA extraction, cDNA synthesis and RT-qPCR

For RT-qPCR analysis, total RNA was extracted from the roots with a Plant Total RNA Mini Kit (Geneaid), according to the manufacturer’s instructions. Total RNA was extracted from 20 mg of lyophilized tissue. The concentration and purity of RNA were determined using a Nanodrop spectrometer (ND-1000, Thermo Fisher Scientific, Massachusetts, USA; https://www.thermofisher.com/il/en/home.html) at 230 nm and 260/280 nm, respectively. Thereafter, RNA integrity was evaluated by agarose gel electrophoresis. cDNA was synthesized from purified RNA using the iScript cDNA Synthesis Kit (Bio-Rad, United States). The generated cDNA was diluted 25 times, and quantitative analysis of transcripts was performed by employing a set of specific primers (see Supplementary Table 1). Melon β-*actin* [ 55] and pumpkin β-*actin* genes were used as reference genes (Supplementary Table 1). For RT-qPCR analysis, 10-μL reaction samples were prepared with 5 μL of Power SYBR® Green PCR Master Mix (Applied Biosystems 7500), 0.3 μL of primers, 2 μL of cDNA and 2.7 μL of DNase-free water. Amplifications were monitored in RT-qPCR using an iCycler IQ multicolor real-time qPCR Detection System (Applied Biosystems 7500). Each plate included a pool of all samples for calibration between runs. Three biological replicates for each sample were normalized to the β-*actin* reference gene (ΔCt = Ct_gene tested_ – Ct β-*actin*). All data were expressed as an n-fold change of gene expression in fruiting vs. fruit-removed plants.

### Statistical analysis

Data were subjected to a two-way ANOVA to test the differences between graft combinations and treatments (fruit removed and fruiting) before ANOVA analysis homogeneity was tested by Levene’s test (*p* > 0.05). If the assumption was not met, transformation (log_2_, log_10_) was performed. When significant differences were scored for a variable, the Tukey–Kramer test was used to determine significant differences between the samples (*p* ≤ 0.05). ANOVA was performed using R package “rstatix.” Pairwise comparison was computed using R package “emmeans.” All statistical analyses were performed using R Statistical Software (ver. 3.2.4).

## Acknowledgments

We thank Ms. Inez Mureinik for editing the manuscript and Mr. Shabait Cohen, Ramat Negev Desert Agro-Research Center for helpful assistance with the melon cultivation. We also thank Ms. Tania Acuna, BGU, for technical support in the lab and field and Ms. Hana Martinková and Ms. Petra Amakorová for their help with phytohormone analyses. This work was partially supported by the Israel Ministry of Agriculture and Rural Development (Eugene Kandel Knowledge Centers) as part of the program ``The Root of the Matter: The Root Zone Knowledge Center for Leveraging Modern Agriculture'' and the ERDF project ``Plants as a tool for sustainable global development'' (No. CZ.02.1.01/0.0/ 0.0/16_019/0000827).

## Author contributions

MD.C., N.TZ., and A.F. conceived and planned the study. MD.C. led and conducted the research. MD.C. designed and conducted greenhouse experiments. MD.C., N.TZ., O.N., and A. F were responsible for data curation. O.N. and A.P. conducted and supervised the hormone analysis. U.Z. and L.Z. assisted with quantitative real-time RT-PCR performance. MD.C. wrote the original draft with N.TZ., A.F. and O.N. All the authors reviewed and approved the final draft.

## Data Availability

The data and materials supporting the conclusions of this study are included (Supplementary information).

## Conflict of interests

The authors declare that they have no conflict of interest.

## Supplementary data

Supplementary data is available at *Horticulture Research* online.

## Supplementary Material

Web_Material_uhac110
