# Supplementary material for: Impairment of root auxin–cytokinins homeostasis induces collapse of incompatible melon grafts during fruit ripening
Source: Hortic Res. 2022 May 17;9:uhac110. doi: 10.1093/hr/uhac110 (PMC9252106; doi:10.1093/hr/uhac110)
Supplement: Web_Material_uhac110 [file web_material_uhac110.zip › Supplementary 1M&M 21-4-22.pptx]

## Slide 1
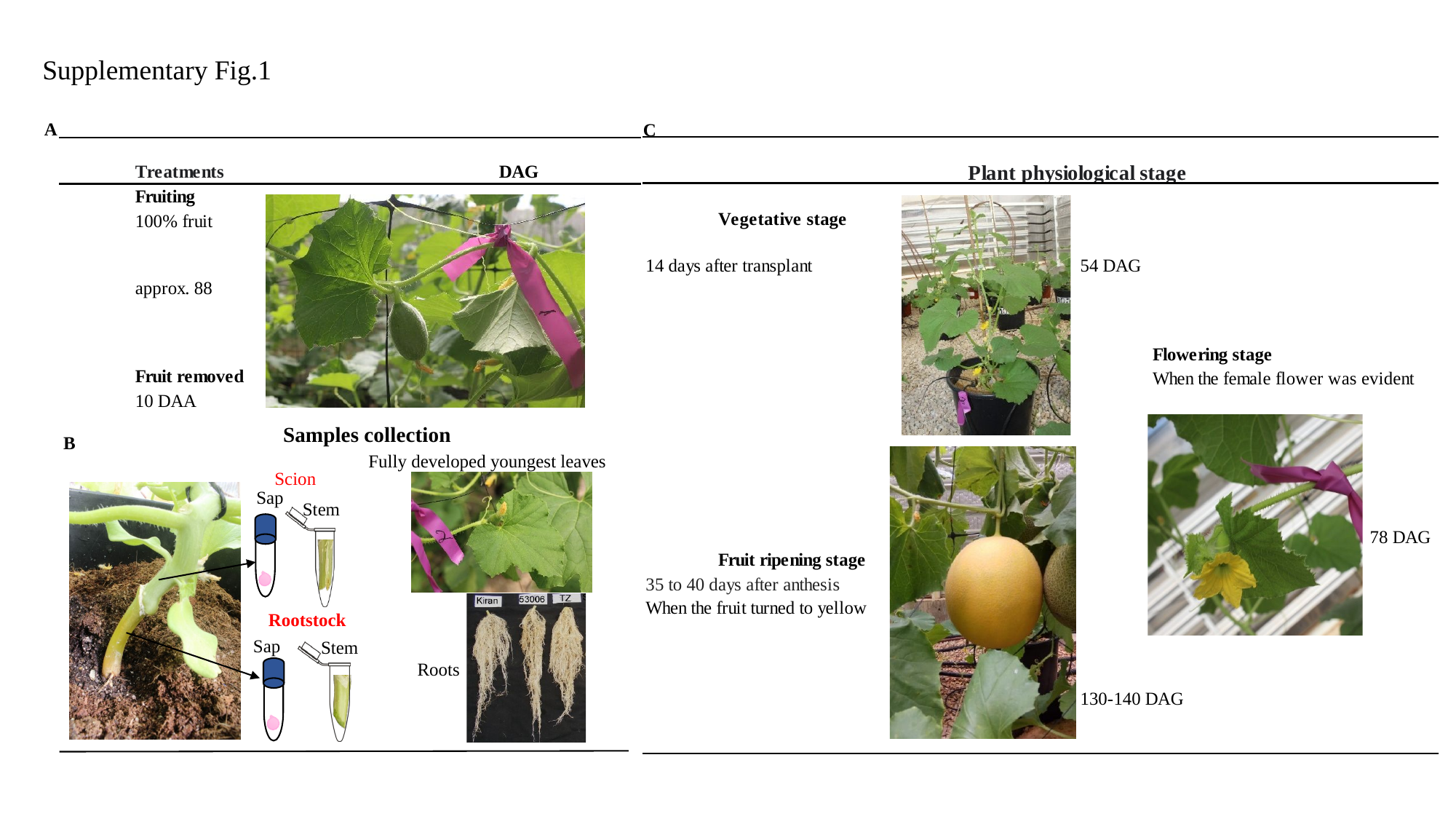

Supplementary Fig.1
A
Samples collection
B
Fully developed youngest leaves
Scion
Sap
Stem
Rootstock
Sap
Stem
Roots
C
