# Supplementary material for: Impairment of root auxin–cytokinins homeostasis induces collapse of incompatible melon grafts during fruit ripening
Source: Hortic Res. 2022 May 17;9:uhac110. doi: 10.1093/hr/uhac110 (PMC9252106; doi:10.1093/hr/uhac110)
Supplement: Web_Material_uhac110 [file web_material_uhac110.zip › Supplementary Figures -clean -21-4-22.docx]

Supplementary Figures

Samples collection


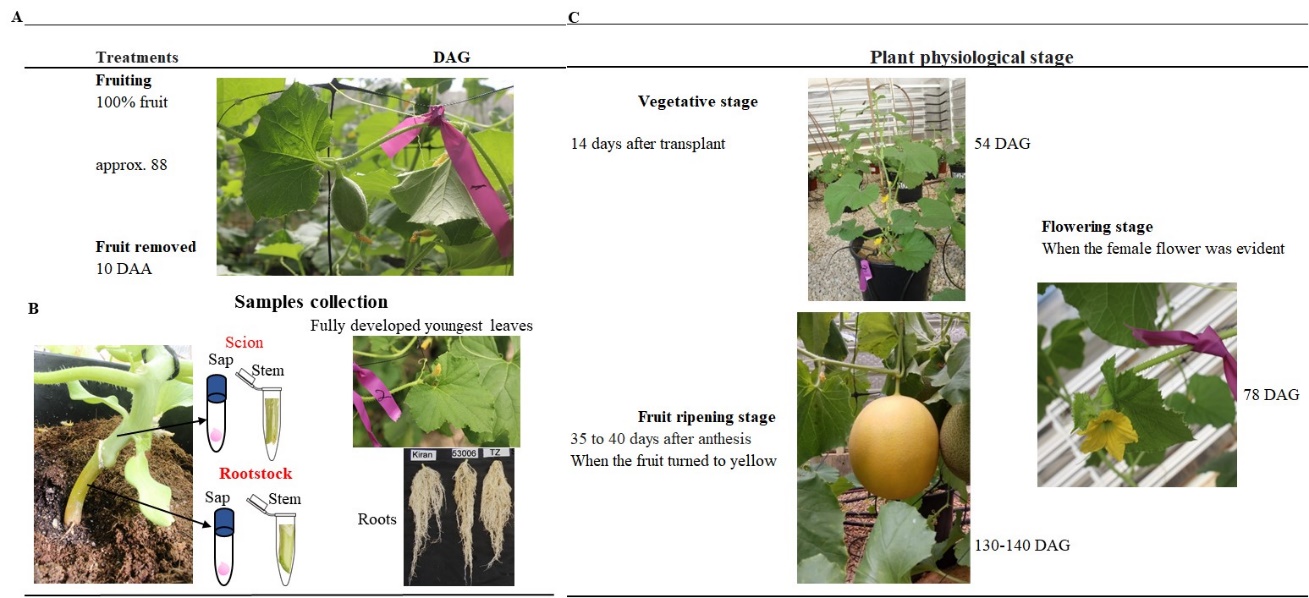


Supplementary Fig. 1 | Summary of the experimental setup. (A) Treatments employed 10 days after anthesis (DAA); (B) illustration of samples collection; and (C) illustration of plant physiological stages (when samples were collected). Notably, only leaf samples were collected at all three stages (vegetative, flowering, and fruit ripening).


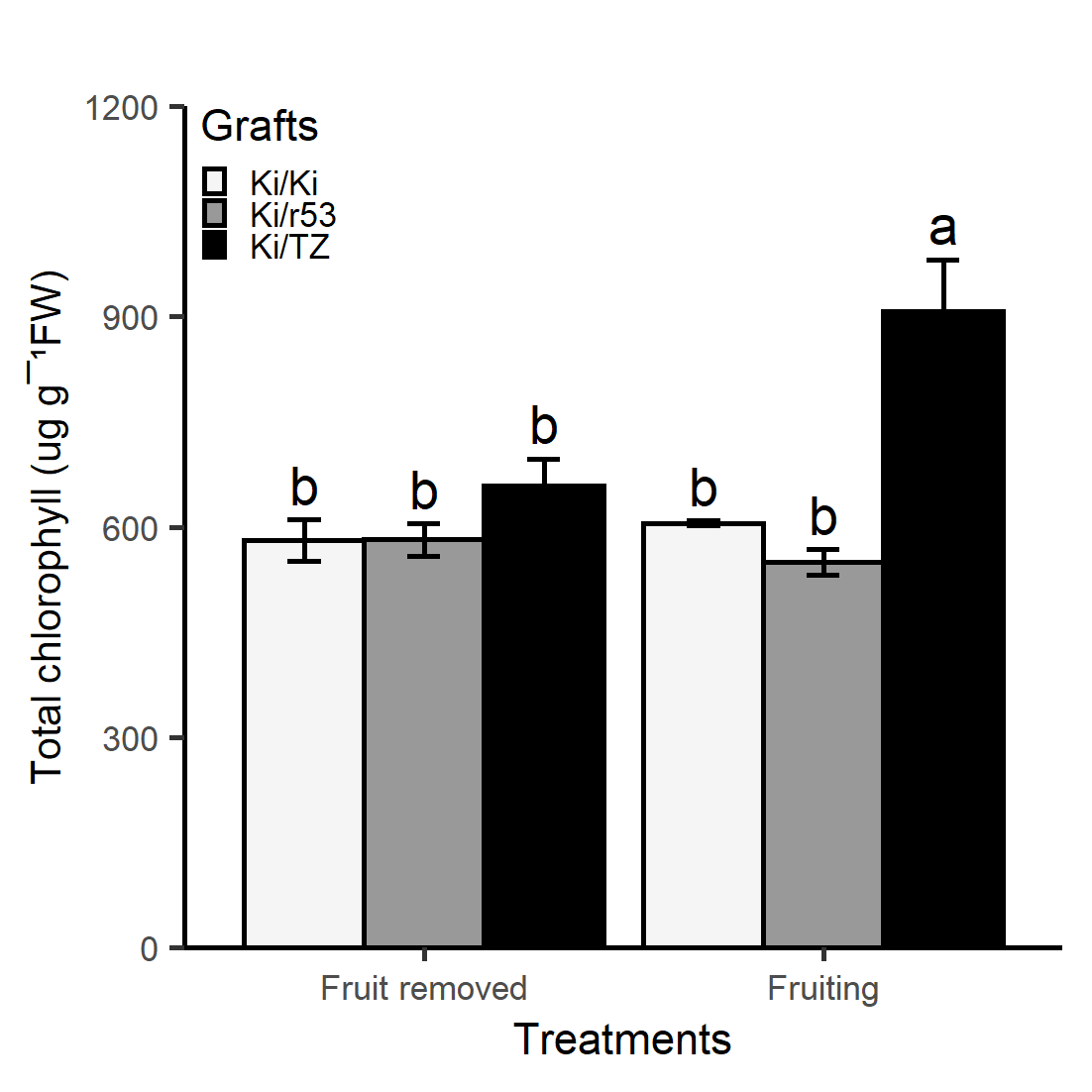


Supplementary Fig. 2 | Leaf total chlorophyll content in Ki/Ki, Ki/r53, and Ki/TZ grafts. Data are means ± s.e. (*n* = 4 biological replicates). Different lowercase letters indicate significant differences evaluated by the Tukey-Kramer multi-comparison test conducted only when a two-way analysis of variance was significant at *p* <0.05.


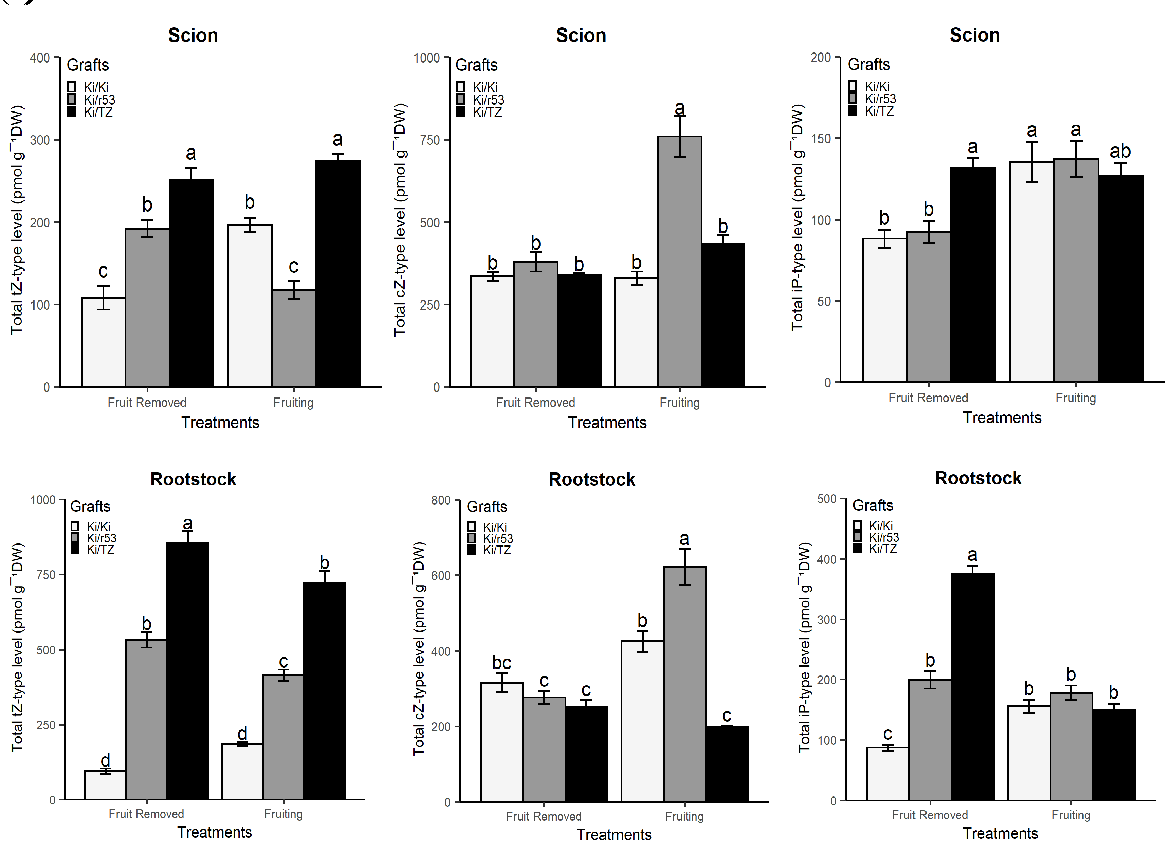


Supplementary Fig. 3 | Scion- and rootstock-sap cytokinin content in Ki/Ki, Ki/r53, and Ki/TZ grafts. (A) Scion sap *trans*-zeatin (*t*Z) type cytokinins; (B) scion sap *cis*-zeatin (*c*Z) type cytokinins; (C) scion sap *N⁶* -isopentenyladenine (iP) type cytokinins; (D) rootstock sap *trans*-zeatin (*t*Z) type cytokinins; (E) rootstock sap *cis*-zeatin (*c*Z) type cytokinins; and (F) rootstock sap *N⁶* -isopentenyladenine (iP) type cytokinins. Data are means ± s.e. (*n* = 4 biological replicates). Different lowercase letters indicate significant differences evaluated by the Tukey-Kramer multi-comparison test conducted only when a two-way analysis of variance was significant at *p* <0.05.


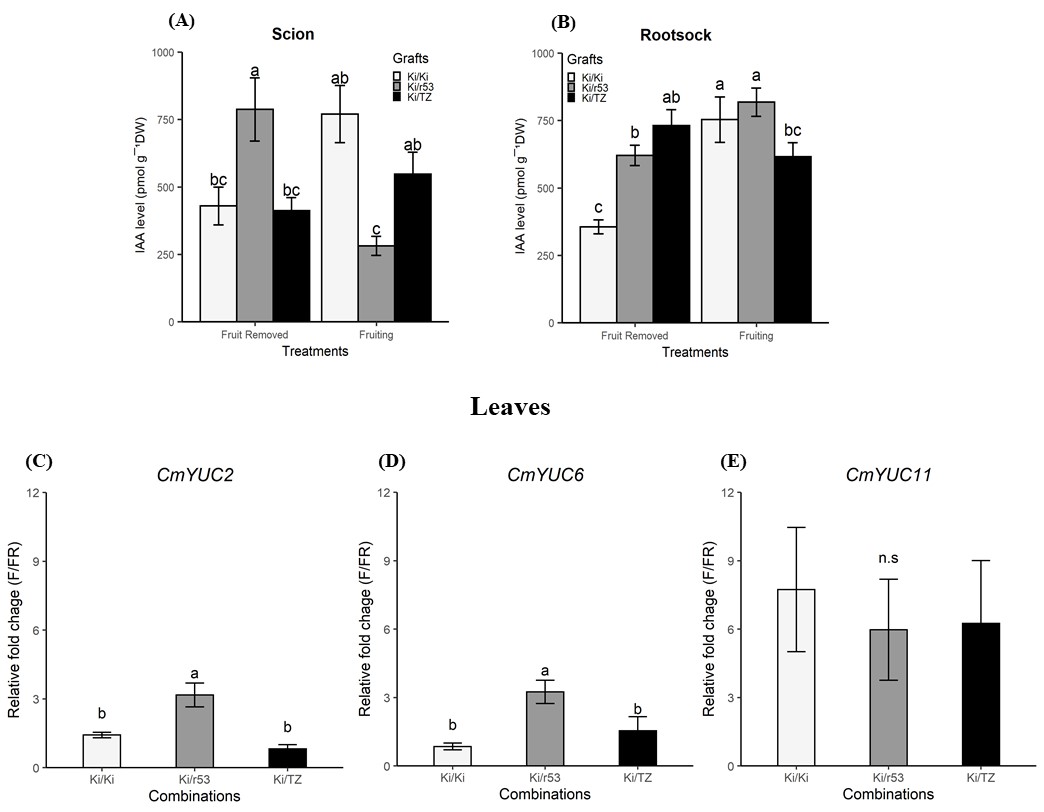


Supplementary Fig. 4 | Sap (scion and rootstock) and quantitative real-time RT-PCR analyses of the fold changes in relative expression levels of *YUCCA* (*YUC*) genes Ki/Ki, Ki/r53, and Ki/TZ under two treatments, i.e., fruiting and fruit removed. (A) Scion sap IAA content; (B) rootstock sap IAA content; (C) leaf *CmYUC2* expression; (D) Leaf *CmYUC6* expression; and (E) leaf *CmYUC11* expression. Data are means ± se (*n* = 4 biological replicates). Bars with different letters indicate significant differences between graft combinations and treatments (fruiting and fruit removed). Two-way ANOVA *P≤* 0.05, as determined by Tukey-Kramer HSD.


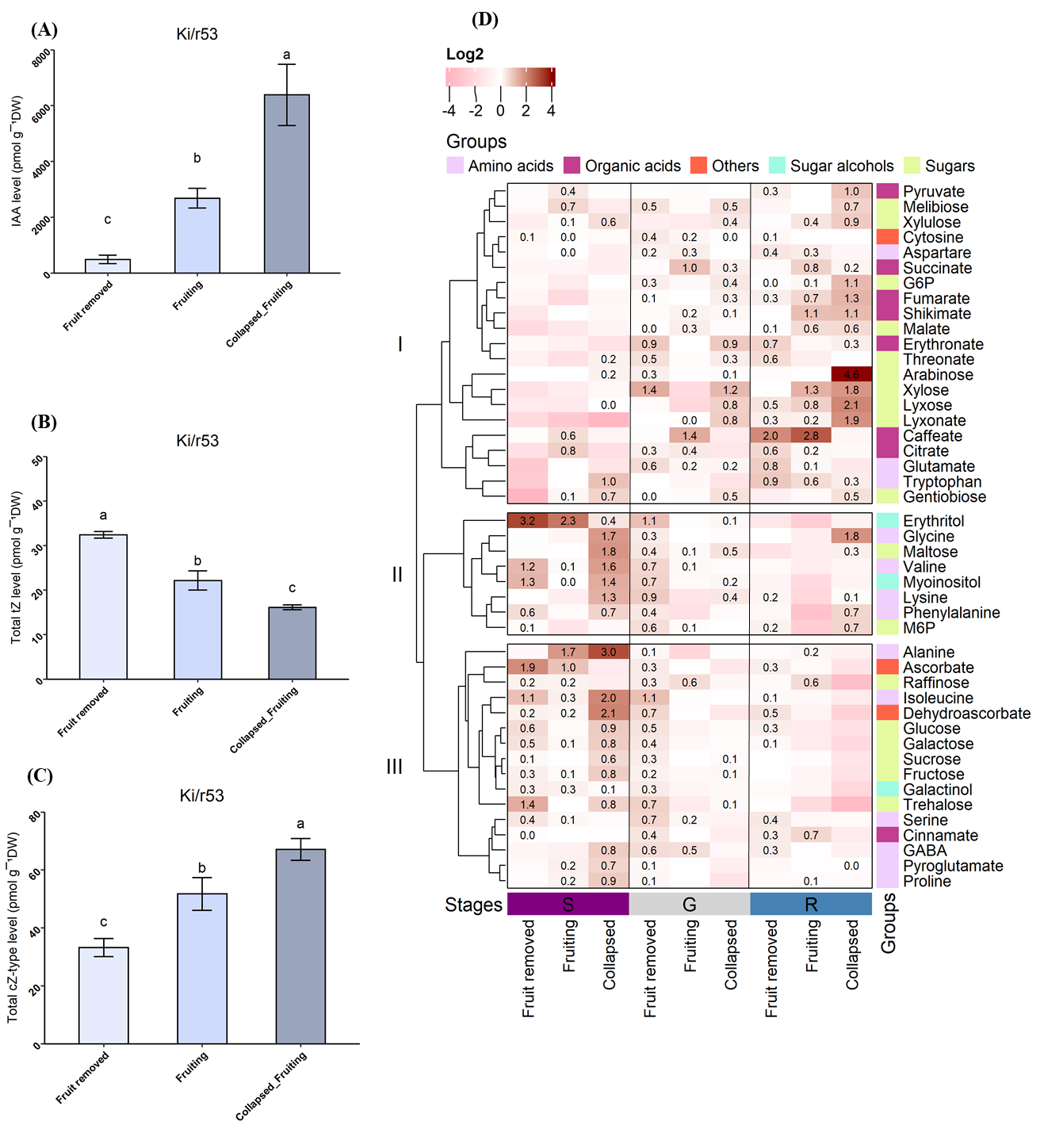


Supplementary Fig. 5 | Root Auxin (IAA), cytokinins content and stem metabolites profiling collected from Ki/r53 grown under three treatments, i.e., fruiting and fruit removed and the collapsed plants**.** (A) Root IAA; (B) root total *t*Z-type cytokinins; (C) root *cZ-*type cytokinins; and (D) heat map showing the hierarchical clustering of the metabolite log_2_ values from the stem (S: scion, G: grafted junction, and R: rootstock). Heat map dark red indicates a high relative abundance of metabolites, and light pink indicates a low relative abundance.
