# Supplementary figures and images for: Impairment of root auxin–cytokinins homeostasis induces collapse of incompatible melon grafts during fruit ripening
Source: Hortic Res. 2022 May 17;9:uhac110. doi: 10.1093/hr/uhac110 (PMC9252106; doi:10.1093/hr/uhac110)

## Slide 1
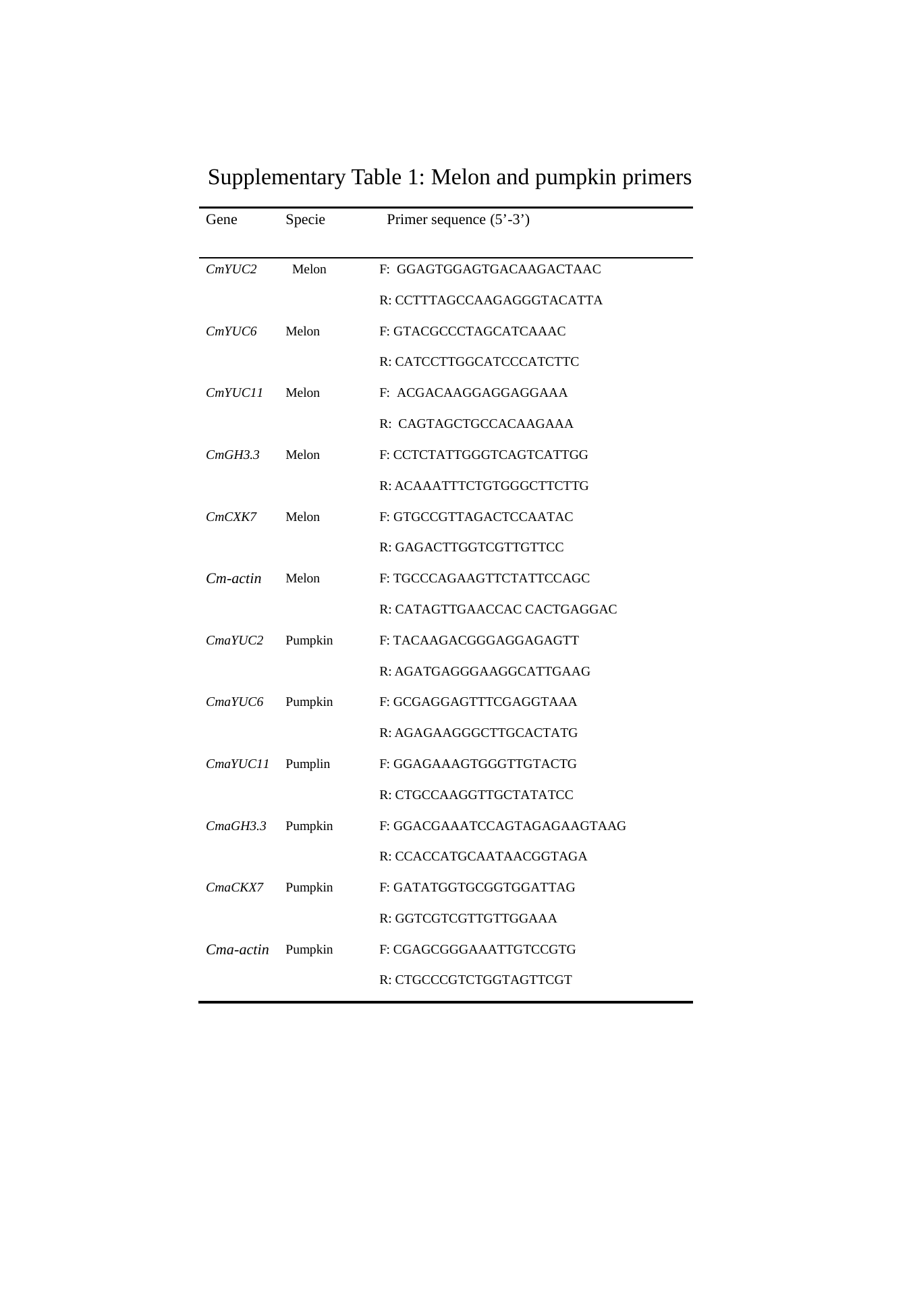

Supplementary Table 1: Melon and pumpkin primers

Supplement: Web_Material_uhac110 [file web_material_uhac110.zip › Supplementary Table 1.pptx]
